# Identifying endoplasmic reticulum stress-related molecular subtypes and prognostic model for predicting the immune landscape and therapy response in pancreatic cancer

**DOI:** 10.18632/aging.205094

**Published:** 2023-10-09

**Authors:** Biao Zhang, Xu Chen, Zhizhou Wang, Fangyue Guo, Xiaonan Zhang, Bingqian Huang, Shurong Ma, Shilin Xia, Dong Shang

**Affiliations:** 1 Department of General Surgery, Clinical Laboratory of Integrative Medicine, The First Affiliated Hospital of Dalian Medical University, Dalian, China; 2 Institute (College) of Integrative Medicine, Dalian Medical University, Dalian, China

**Keywords:** pancreatic cancer, endoplasmic reticulum stress, prognostic model, tumour microenvironment, drug sensitivity

## Abstract

Endoplasmic reticulum stress (ERS) is caused by the accumulation of intracellular misfolded or unfolded proteins and is associated with cancer development. In this study, pan-cancer analysis revealed complex genetic variations, including copy number variation, methylation, and somatic mutations for ERS-related genes (ERGs) in 33 kinds of cancer. Consensus clustering divided pancreatic cancer (PC) patients from TCGA and GEO databases into two ERS-related subtypes: ERGcluster A and B. Compared with ERGcluster A, ERGcluster B had a more active ERS state and worse prognosis. Subsequently, the ERS-related prognostic model was established to quantify the ERS score for a single sample. The patient with a low ERS score had remarkably longer survival times. ssGSEA and CIBERSORT algorithms revealed that activated B cells and CD8+ T cells had higher infiltration in the low ERS score group, but higher infiltration of activated CD4+ T cells, activated dendritic cells, macrophages, and neutrophils in the high ERS score group. Drug sensitivity analysis indicated the low ERS score group had a better response to gemcitabine, paclitaxel, 5-fluorouracil, oxaliplatin, and irinotecan. RT-qPCR validated that MET, MUC16, and KRT7 in the model had higher expression levels in pancreatic tumour tissues. Single-cell analysis further revealed that MET, MUC16, and KRT7 were mainly expressed in cancer cells in PC tumour microenvironment. In all, we first constructed the ERS-related molecular subtypes and prognostic model in PC. Our research highlighted the vital role of ERS in PC and contributed to further research on molecular mechanisms and novel therapeutic strategies for PC in the future.

## INTRODUCTION

Pancreatic cancer (PC) is one of the foremost common malignant tumours in the digestive system, with the characteristics of insidious onset, rapid progression, and poor prognosis; therefore, the 5-year survival rate is simply 2%-9% [1]. In the United States, PC was the third leading explanation for tumour-related death, with an estimated 62,210 new cases and 49,830 deaths of PC in 2022 [2]. By 2030, PC is anticipated to rank as the second most common cancer-related cause of death [3]. In recent years, the treatment and prognosis of PC have not significantly improved. Surgery combined with adjuvant systemic chemotherapy is the mainstay for PC. However, because of the shortage of typical symptoms within the early stage of PC, most patients square measure already within the middle and advanced stages after they present with symptoms and thus lose the opportunity for radical surgery; only 10%-20% of patients can receive surgical treatment [4]. Chemotherapy remains the primary treatment option for patients with metastatic PC. But even with first-line chemotherapy regimens like FOLFIRINOX (fluorouracil, oxaliplatin, irinotecan, and leucovorin) or gemcitabine combined with nab-paclitaxel, the response rate for PC is only about 30% [5]. In recent years, targeted therapy and immunotherapy have created great progress, revolutionizing the treatment of cancers like hepatocellular carcinoma, non-small cell lung cancer, and nephritic cell carcinoma [6–9]. However, targeted therapy and immunotherapy have not achieved the desired effect for PC due to the complex, highly immunosuppressive tumour microenvironment [10, 11]. Hence, it's still important and imperative to explore the mechanisms of PC development as well as find effective treatment methods.

Endoplasmic reticulum is the central organelle that functions in protein synthesis, folding, and modification. Although this process is finely regulated, a variety of cellular internal and external factors can disrupt the ability of endoplasmic reticulum to fold proteins and trigger endoplasmic reticulum stress (ERS) characterized by the accumulation of misfolded or unfolded proteins [12]. Tumour cells are often exposed to various factors that affect protein homeostasis (e.g., hypoxia, nutrient deficiency, acidosis, oncogenic activation, changes in chromosome number, and increased secretory capacity), resulting in persistent ERS, which ultimately affects tumour cell function and survival [13]. In response to ERS, cells activate a range of adaptive mechanisms to enhance folding and clearance and restore protein balance within the endoplasmic reticulum, called the unfolded protein response (UPR) [14]. UPR can favour cell survival, help reduce the buildup of misfolded proteins, and restore the function of the endoplasmic reticulum. If ERS cannot be addressed, the UPR signal will switch from survival to pro-apoptosis [15]. In mammalian cells, UPR is initiated primarily by three endoplasmic reticulum transmembrane proteins, including IRE1α, PERK, and ATF6 [14, 16]. Many studies have demonstrated that UPR activation is associated with many characteristics of cancer, including angiogenesis, genomic instability, cell proliferation, invasion and dormancy, chemotherapy resistance, and tumour immunity [13, 17]. Pereira et al. [18] found that positive angiogenic regulatory factors (including VEGFA, FGF2, angiogenin, and IL8) were significantly upregulated during ERS, and activation of UPR could promote the expression of VEGFA mRNA and protein more effectively than hypoxia, thus promoting angiogenesis. Study from Cubillos-Ruiz et al. [19] showed that activation of ERS response factor XBP1 induced the biosynthesis of triglycerides in tumour-associated dendritic cells, resulting in abnormal lipid accumulation and inhibition of the ability of tumour-associated dendritic cells to support anti-tumour T cells, thereby promoting the progression of ovarian cancer. Adjuvant therapies such as chemotherapy and targeted therapy are important means to improve the survival time for cancer patients. However, their effectiveness is challenged by multiple drug resistance mechanisms formed by tumour cells before and during treatment, including reduced drug uptake, drug target change, repair of drug-induced harm, and unfitness to drug-induced cell death [20]. Salaroglio et al. [21] showed that PERK mediated Nrf2-driven MRP1 transcription, thereby inducing tumour cell resistance to ERS and chemotherapy. Presently, the specific mechanism of ERS in PC has not been comprehensively studied. Exploring the molecular characteristics and roles of endoplasmic reticulum stress-related genes (ERGs) in PC may help find new strategies for diagnosis and treatment.

Based on this evidence, we first identified the ERS-related molecular subtypes and prognostic signature for PC by comprehensive bioinformatics analysis and *in vitro* and *in vivo* experiments.

## MATERIALS AND METHODS

### Data acquisition and preprocessing

The RNA expression data as well as clinical information (containing 185 cases of PC samples) were acquired via The Cancer Genome Atlas (TCGA) platform (https://portal.gdc.cancer.gov/). In addition, the copy number variation (CNV), methylation and somatic mutation data of thirty-three kinds of cancer from TCGA database were acquired to explore the genetic variations for ERGs in pan-cancer. GSE28735 (containing 45 cases of PC), GSE62452 (containing 69 cases of PC), GSE57495 (containing 63 cases of PC) and GSE85916 (containing 80 cases of PC) datasets were acquired via the Gene Expression Omnibus (GEO) platform (https://www.ncbi.nlm.nih.gov/geo/). Genotype-Tissue Expression Project (GTEx) (including 167 cases of pancreatic tissue from healthy people) was acquired via the UCSC Xena platform (https://xenabrowser.net/datapages/). Using the “sva” package to get rid of batch effects among totally different datasets [22, 23]. Samples with a survival time of less than 30 days were excluded for survival analysis. ERGs were acquired through the Molecular Signatures Database (MSigDB, http://www.gsea-msigdb.org/) [24]. Differentially expressed genes (DEGs) were identified between the pancreatic tumour and normal tissue from TCGA and GTEx datasets by using the “limma” R package with the filter condition of |log2 fold change (FC)| > 1 as well as adjusted p-values < 0.05. Then, the intersection of DEGs and ERGs was identified for follow-up analysis.

### Consensus clustering

We first combined all the PC patients from the TCGA, GSE28735, GSE62452, GSE57495 and GSE85916 datasets, then used the "ConsensusClusterPlus" package to conduct an unsupervised cluster analysis based on the differentially expressed ERGs. The optimal cluster number was determined using the consensus matrix and the cumulative distribution function (CDF) curve. Every patient could be grouped into different subtypes (ERGcluster A and B). The principal component analysis (PCA) with good dimensionality reduction ability was used to illustrate the consistency of the cluster. Survival times between different subtypes were compared utilizing Kaplan-Meier curve. Gene set variation analysis (GSVA) as well as gene set enrichment analysis (GSEA) were performed to compare the differences in biological behaviour in different subtypes [25]. Single-sample gene set enrichment analysis (ssGSEA) was performed to explore the variations of pancreatic tumour immune microenvironment between different subtypes.

### Differential expression analysis between the different ERS-related subtypes

To analyze the differences between the different ERS-related patterns, we utilized the “limma” package to spot the ERS-related DEGs between different subtypes. Then we performed univariate Cox regression to assess the prognostic value of the ERS-related DEGs, and the p-value < 0.05 was considered statistically significant. The Gene Ontology (GO) and Kyoto Encyclopedia of Genes and Genomes (KEGG) enrichment analyses were performed to explore the enriched pathways for the ERS-related DEGs, and the filter’s parameters was set to a q-value < 0.05 [26]. What’s more, the “ConsensusClusterPlus” package was utilized to perform unsupervised cluster analysis based on the expression of ERS-related DEGs, and each patient could be classified into a type of geneCluster A or B.

### Construction of the ERS-related prognostic model

For further quantifying the ERS score in a single sample, we used LASSO and multivariate Cox regression analyses to establish an ERS-related prognostic model for PC based on the ERS-related DEGs. Utilizing the “caret” package to randomly divide all PC samples into training cohorts and verification cohorts in a ratio of 5:5. The ERS score of every sample could be computed through the model formula, while each sample was grouped into high or low ERS score by comparing it to the median value of ERS score in the training cohort.

### Enrichment analysis and immune analysis

GSVA as well as GSEA were performed to compare the differences in the enriched pathways in the different ERS score groups [25]. To further explore the variations of pancreatic tumour immune microenvironment between different clusters and between different ERS score groups, ssGSEA was performed to compute a total of 28 immune cell infiltration scores for each sample through the “GSVA” and “GSEABase” packages. What’s more, the proportions of 22 immune cells were computed for each sample by CIBERSORT algorithm [27]. The p-value < 0.05 represented that the infiltration assessment of 22 immune cell subtypes for PC was accurate, and these samples were utilized to further analysis.

### Mutation analysis and drug sensitivity analysis

Utilizing the “maftools” package to investigate and visualize the mutation landscape in both high and low ERS score groups. 38Mb is routinely extracted according to the length of human exons, so tumour mutation burden (TMB) per sample was calculable to be up to the whole mutation frequency divided by 38 Mb [28]. Subsequently, the association between ERS score and TMB was further evaluated.

The “OncoPredict” package has a predictive effect on *in vivo* drug responses for patients with malignant tumours [29]. It can fit the gene expression data to the semi-maximum inhibitory concentration of neoplastic cell lines for medication in Genomics of Drug Sensitivity in Cancer (https://www.cancerrxgene.org/). The “OncoPredict” package was utilized to assess the variations in drug sensitivity between different PC patients. For further assessing the correlation between model gene and drug sensitivity, we downloaded relevant gene expression data and Food and Drug Administration approved drug sensitivity data from the CellMiner database (https://discover.nci.nih.gov/cellminer/) [30].

### Gene expression analysis and single-cell analysis

GEPIA database (http://gepia.cancer-pku.cn/) is an open platform that can analyze gene expression difference, correlation, and prognostic value [31], which was employed for investigating gene expression variations between pancreatic tumour and normal tissues. The Human Protein Atlas (HPA) (version 22.0 https://www.proteinatlas.org/) is a public platform designed for creating maps of protein expression patterns [32], which was employed for investigating the distribution and expressed variations of genes in the model. Single-cell RNA-seq offers transcription data from a single cell. The Tumour Immune Single-Cell Hub (TISCH) database (http://tisch.comp-genomics.org) is a single-cell RNA-seq platform immersed in tumour microenvironment [33, 34], which was employed for investigating the proportion of each cell subset in the pancreatic tumour.

### Cell lines and tissues

HPDE6-C7, a human pancreatic ductal epithelium cell line, was obtained through the American Type Culture Collection (ATCC, Manassas, VA, USA). Human PC cell lines (CF-PAC1 as well as Panc-1) were obtained from Procell Life Science and Technology Co., Ltd. And KeyGEN BioTECH (Jiangsu province, China) supplied the human PC cell line BxPC-3. Seven pairs of pancreatic tumour tissues and paracancer tissues were obtained from PC patients operated on at the First Affiliated Hospital of Dalian Medical University. All acquired tissues are immediately flash-frozen in liquid nitrogen and kept at -80° C. Informed consent was obtained from the patients participating in this study.

### Real-time quantitative PCR

Total RNAs were extracted from cell lines and tissues by an extraction reagent, TRIzol (Accurate Biotechnology, Shanghai, China). Subsequently, reverse transcription was performed to acquire cDNAs. Finally, real-time quantitative PCR (RT-qPCR) was conducted using SYBR® Green Premix Pro Taq HS qPCR Kit (Accurate Biotechnology, Shanghai, China). And GAPDH was used as a control reference, utilizing ΔΔCt technique for quantifying RNA expression. We acquired primer sequences through GenePharma (Suzhou, China), for human KRT7 (Forward: 5’-CCAGGAGGAGAGCGAGCAGATC-3’; Reverse: 5’-GCAGCAGCGTCCACTTGGTC-3’), MUC16 (Forward: 5’-CATTACCAGCAAGTAGCCACTCCTC-3’; Reverse: 5’-CGTCCAACACCTCAGTAGTCTTCAC-3’); MET (Forward: 5’-GTCCTATGGCTGGTGGCACTTTAC-3’; Reverse: 5’-TGGTTTGGGCTGGGGTATAACATTC-3’).

### Statistical analysis

R (version 4.1.2) and GraphPad Prism 9 were utilized for statistical analysis and visualization in the current investigation. For metric data with a normal distribution, utilizing t-test to perform the differential analysis. For metric data without a normal distribution, Wilcoxon’s rank sum test was utilized for comparison between two groups, and the Kruskal-Wallis test was utilized for comparison among multiple groups. Utilizing the Spearman or Pearson test to perform the correlation analysis. Utilizing the Kaplan–Meier method to perform survival analysis. A p-value < 0.05 represented statistical significance.

## RESULTS

### Identification and pan-cancer analysis of ERGs

**Figure 1:**
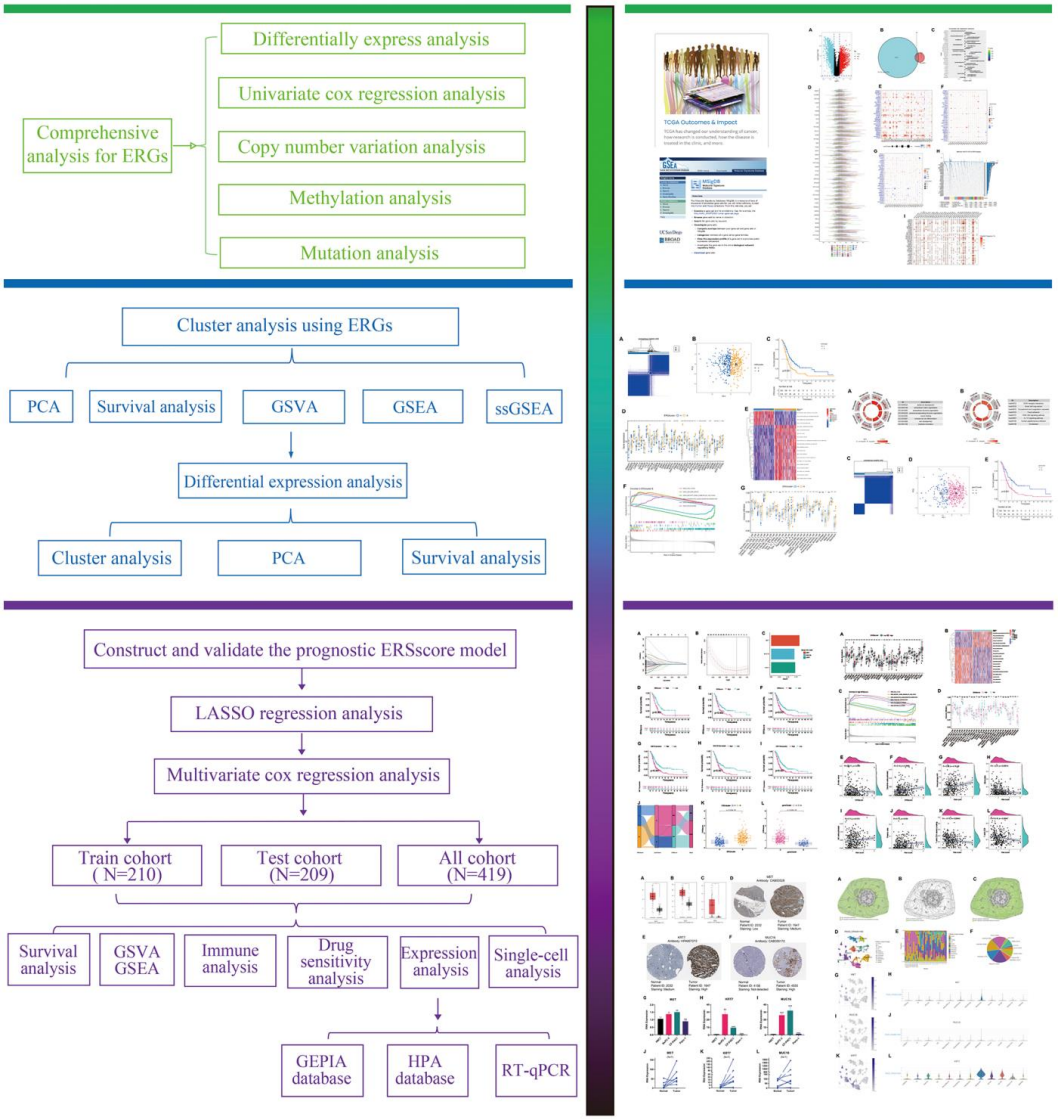
The flowchart of this study.

**Figure 2:**
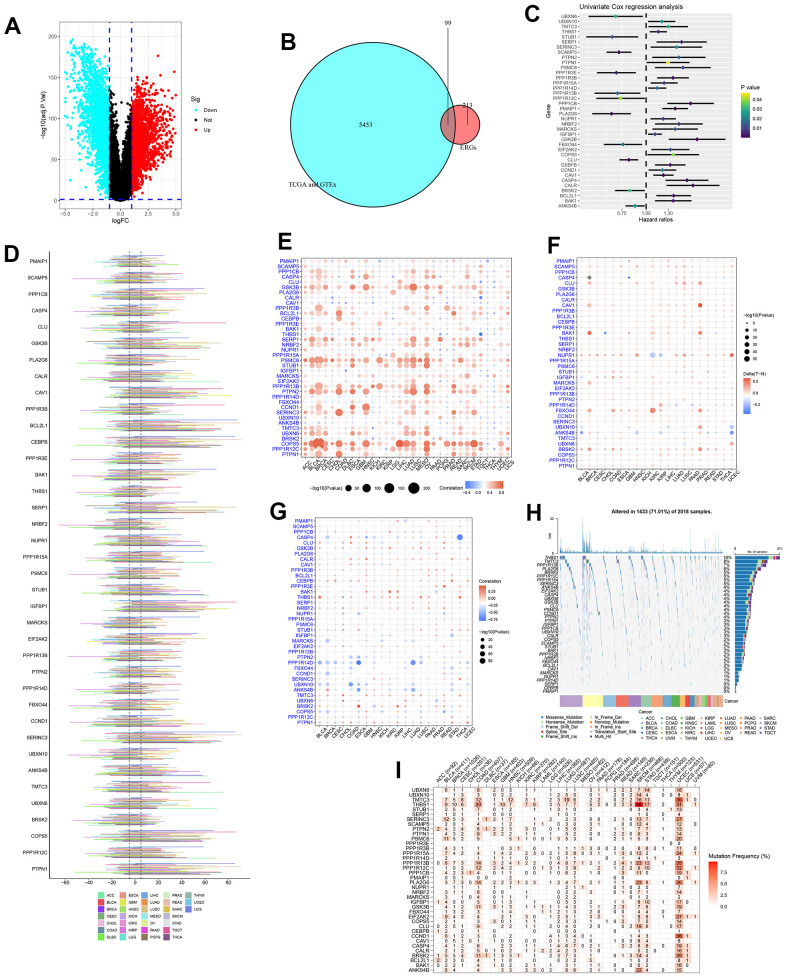
**Identification and pan-cancer analysis of endoplasmic reticulum stress-related genes (ERGs).** (**A**) The differentially expressed genes (DEGs) between pancreatic tumours and normal tissues. (**B**) The intersection of DEGs and ERGs. (**C**) Univariate Cox regression analysis for ERGs. (**D**) The copy number variation (CNV) of ERGs within pan-cancer. (**E**) The correlation between CNV and expression. (**F**) Methylation difference of ERGs between tumour and normal tissues. (**G**) The correlation between methylation and expression. (**H**) The oncoplot of ERGs mutation within pan-cancer. (**I**) Mutation frequency of ERGs.

The process of this study was shown in Figure 1. Based on TCGA and GTEx databases, we obtained 5552 DEGs in tumour tissue and normal tissue of the pancreas (Figure 2A). 15 endoplasmic reticulum stress-related gene sets with a total of 660 genes were obtained from MSigDB (Supplementary Table 1). After 348 duplicate genes were removed, 312 ERGs remained (Supplementary Table 2). Then the intersection of DEGs and ERGs was used to acquire 99 differentially expressed ERGs (Figure 2B). The univariate Cox regression analysis revealed 38 ERGs were related to the prognosis of PC (Figure 2C).

The genetic changes of 38 ERGs in pan-cancer were further assessed. And the results found the CNV frequency of ERGs was generally more than 5% in most cancer types except thyroid carcinoma (THCA), the CNV types of genes CAV1, BCL2L1, CEBPB, IGFBP1, SERINC3, COPS5, and PTPN1 were mainly amplification; the CNV types of gene BRSK2 were mainly deletion; and the CNV types of most ERGs in uterine carcinosarcoma (UCS) and testicular germ cell tumors (TGCT) were mainly amplification, which was the opposite trend in diffuse large B-cell lymphoma (DLBC) and kidney renal papillary cell carcinoma (KIRP) (Figure 2D). The CNV and expression levels of ERGs showed a remarkably positive correlation in most cancer types. For instance, the expression of COPS5 has a remarkably positive correlation with CNV in bladder urothelial carcinoma (BLCA), lower grade glioma (LGG), skin cutaneous melanoma (SKCM) (Figure 2E). These revealed that the CNV for ERGs was a common genetic alteration and was associated with gene expression within most tumours. Besides, DNA methylation could also affect gene expression and be linked to carcinogenesis. We analysed the methylation difference between tumour and normal tissues, and results manifested the most ERGs were hypermethylated within most malignancies, while ANKS4B was hypomethylated within most cancers types (Figure 2F). We found a complex relationship between gene methylation and expression. The relationship between methylation and expression of genes GSK3B, THBS1, and PTPN1 manifested a positive correlation in most tumour types, whereas this was reversed for genes PPP1R14D (Figure 2G). Finally, the mutation of ERGs in pan-cancer was evaluated. The most common type of genetic mutation was missense mutation, and the top four ERGs with the highest mutation frequency were THBS1 (10%), TMTC3 (8%), PPP1R13B (7%), and PLA2G6 (6%) (Figure 2H). The top four cancer types with a relatively high mutation frequency were SKCM, uterine corpus endometrial carcinoma (UCEC), colon adenocarcinoma (COAD) and stomach adenocarcinoma (STAD) (Figure 2I).

### Identification of ERS-related molecular subtypes

**Figure 3:**
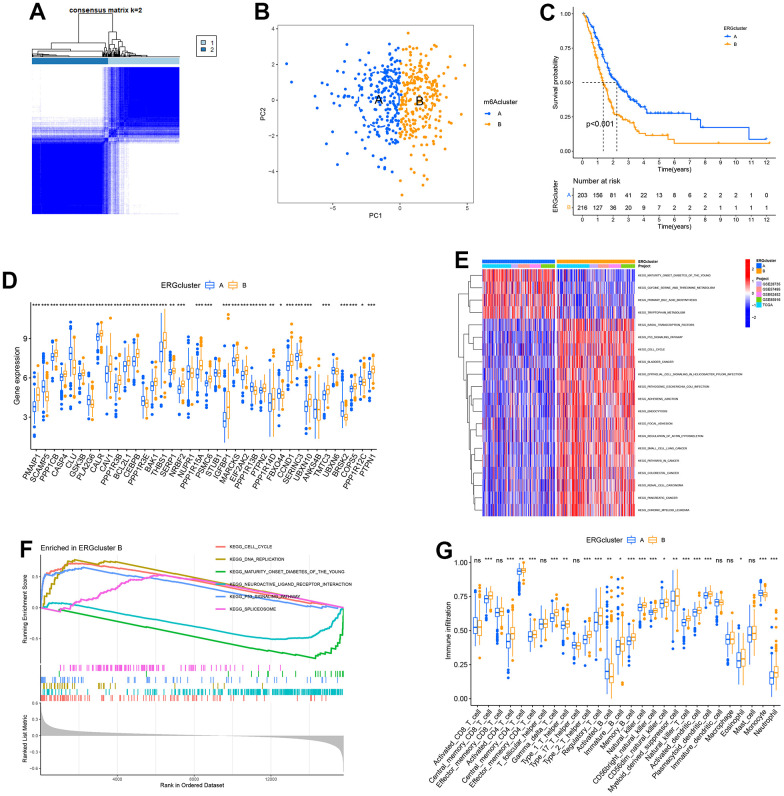
**Identification of endoplasmic reticulum stress (ERS)-related molecular subtypes.** (**A**) Heatmap of consensus matrix when k = 2. (**B**) The principal component analysis could remarkably distinguish ERGcluster A and B. (**C**) The survival curve of ERGcluster A and B. (**D**) The expression difference of endoplasmic reticulum stress-related genes (ERGs) between ERGcluster A and B. (**E**) GSVA analysis. (**F**) GSEA analysis. (**G**) ssGSEA analysis. (*p<0.05; **p<0.01; ***p<0.001).

All samples in the TCGA, GSE28735, GSE62452, GSE57495 and GSE85916 datasets were pooled, and then unsupervised cluster analysis was performed to identify the ERS-related molecular feature for PC. The CDF curve indicated the optimal cluster number was 2 (Supplementary Figures 1, 2). All samples were classified into two clusters: ERGcluster A and B (Figure 3A). PCA could clearly distinguish PC patients with different ERS-related molecular features (Figure 3B). Survival analysis revealed that the prognosis of ERGcluster A was considerably better than that of ERGcluster B (Figure 3C). Most ERGs were highly expressed in ERGcluster B, indicating that ERGcluster B may have a higher ERS level (Figure 3D).

GSVA showed that the remarkable enrichment pathways in ERGcluster A included “primary bile acid biosynthesis”, “tryptophan metabolism”, “maturity onset diabetes of the young”, etc. The remarkable enrichment pathways in ERGcluster B contained “basal transcription factors”, “p53 signaling pathway”, “cell cycle”, “focal adhesion”, “pathways in cancer”, and “pancreatic cancer”, and these enrichment pathways may be associated with the poorer prognosis in ERGcluster B (Figure 3E). GSEA suggested that “cell cycle”, “DNA replication”, “p53 signaling pathway” and “spliceosome” were remarkably enriched in ERGcluster B, “maturity onset diabetes of the young”, and “neuroactive ligand receptor interaction” were remarkably enriched in ERGcluster A (Figure 3F), which was consistent with the GSVA result.

For understanding the variations of the tumour immune microenvironment in different ERS-related molecular features, ssGSEA was carried out. The results indicated that the infiltration level in activated B cells and monocytes was significantly higher in ERGcluster A, and activated CD4+ T cells, activated dendritic cells (DC), immature B cells, natural killer (NK) cells, neutrophils, regulatory T cells (Tregs), myeloid-derived suppressor cells (MDSCs), and type 1 and type 2 T helper cells had a significantly higher infiltration score in ERGcluster B (Figure 3G).

### Differentially expressed analysis in the different ERS-related molecular subtypes

**Figure 4:**
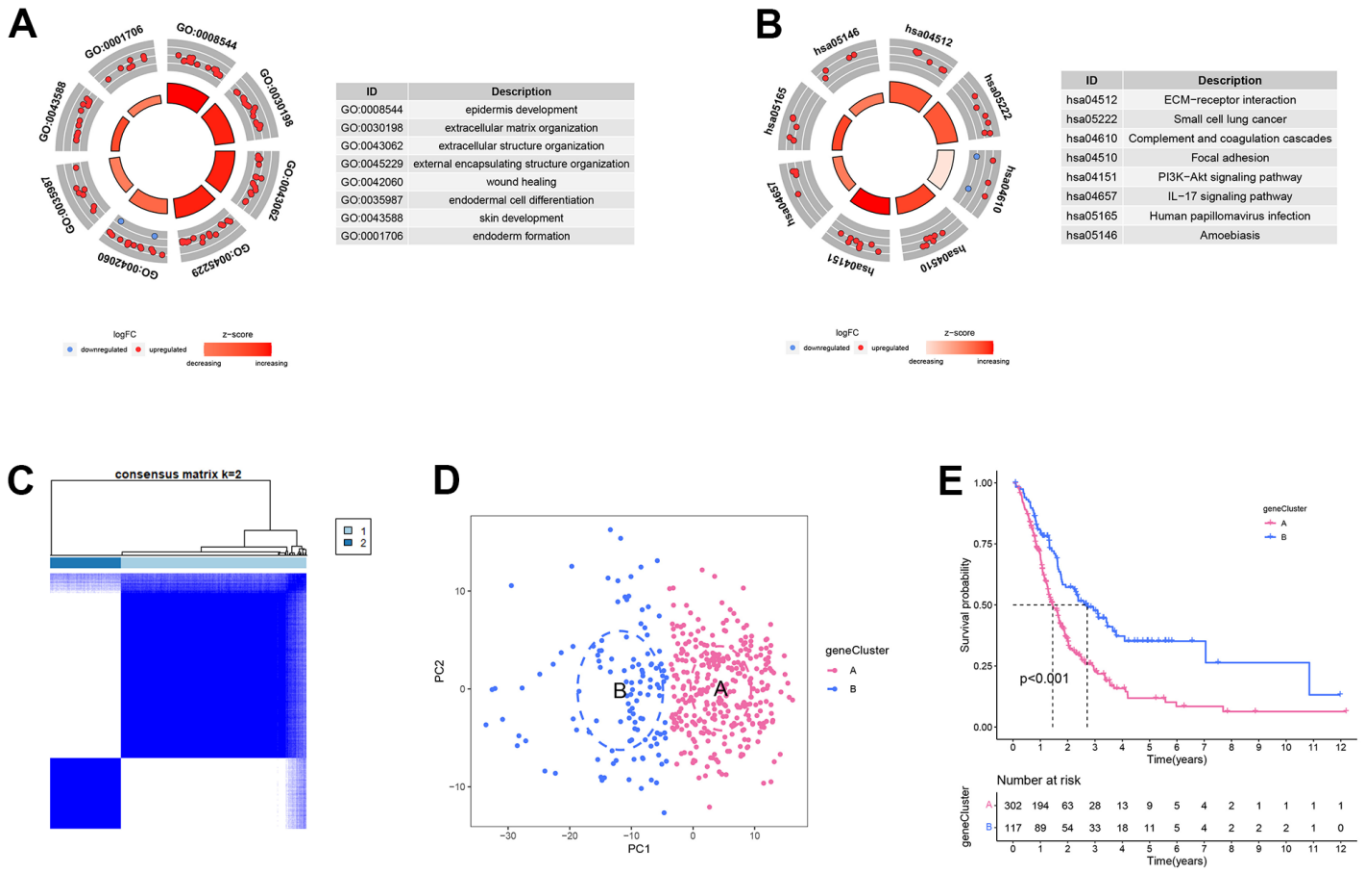
**Differentially expressed analysis in the different endoplasmic reticulum stress-related subtypes.** (**A**) GO enrichment analysis. (**B**) KEGG enrichment analysis. (**C**) Heatmap of consensus matrix when k = 2. (**D**) The principal component analysis could remarkably distinguish geneCluster A and B. (**E**) The survival curve of geneCluster A and B.

For further exploring the variations between different ERS-related molecular subtypes, we screened 164 ERS-related DEGs between ERGcluster A and B (Supplementary Figure 3). Univariate Cox regression revealed 99 ERS-related DEGs were related to the prognosis in PC (Supplementary Figure 4). GO enrichment analysis showed the 99 ERS-related DEGs were considerably enriched in “epidermis development”, “extracellular matrix organization”, “wound healing”, “skin development”, etc. (Figure 4A). KEGG pathway enrichment analysis indicated the 99 ERS-related DEGs were considerably enriched in “ECM−receptor interaction”, “Complement and coagulation cascades”, “PI3K−Akt signaling pathway”, “IL−17 signaling pathway”, etc. (Figure 4B). Subsequently, consensus clustering analysis was carried out based on the 99 ERS-related DEGs, and all samples were classified into two clusters: geneCluster A and B (Figure 4C). PCA could clearly distinguish the different patients between geneCluster A and B (Figure 4D). Survival analysis indicated the prognosis of geneCluster A was remarkably worse than geneCluster B (Figure 4E).

### Establishment of ERS-related prognostic model

**Figure 5:**
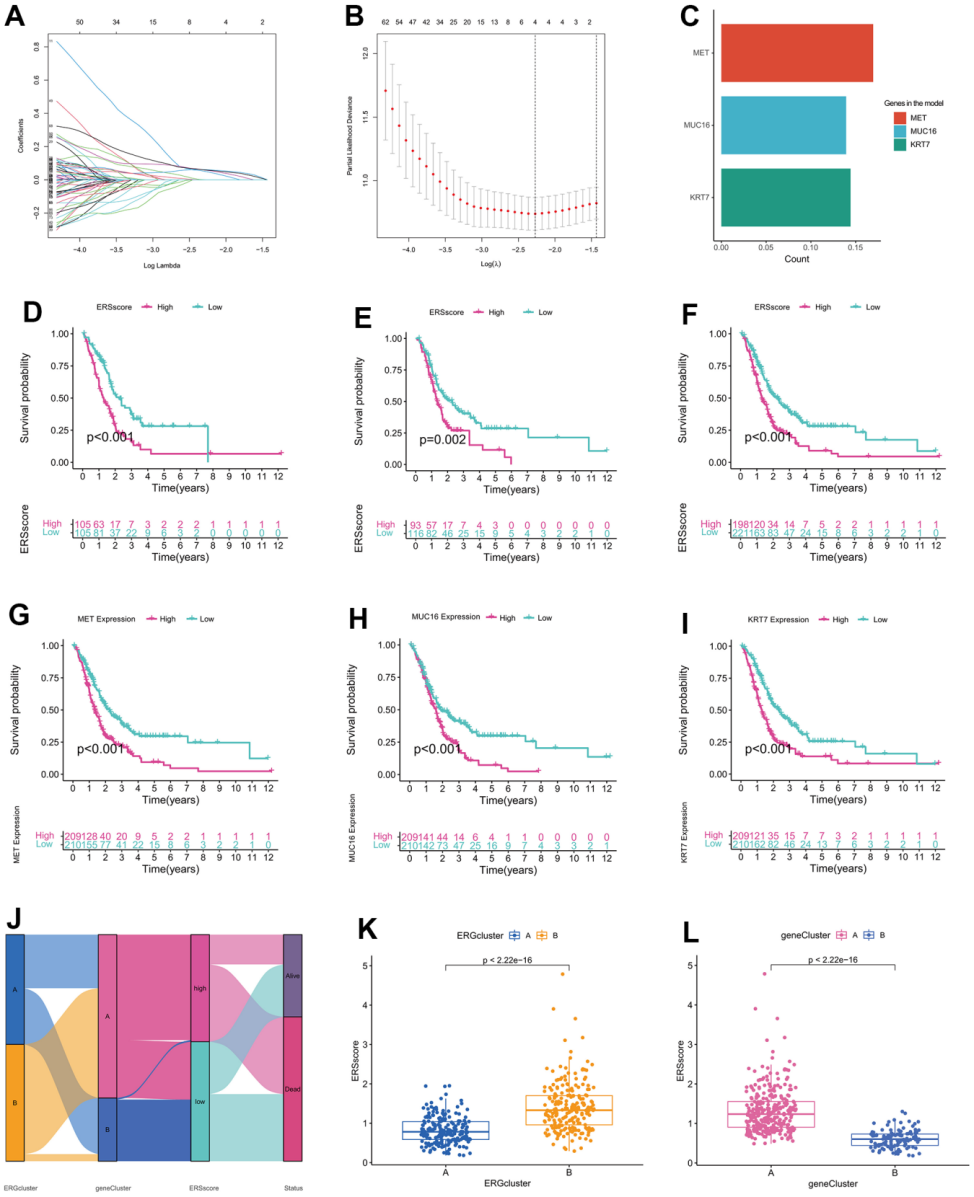
**Establishment of endoplasmic reticulum stress-related prognostic model.** (**A**) Coefficient path diagram for LASSO regression. (**B**) The cross-validation curve. (**C**) Coefficients of MET, MUC16, and KRT7 in the model. Survival curve of the training cohort (**D**), validation cohort (**E**), and whole cohort (**F**). Survival curve between high-expression and low-expression groups of MET (**G**), MUC16 (**H**), and KRT7 (**I**). (**J**) Alluvial diagram of changes in ERGclusters, geneClusters, ERS score and survival state. (**K**) The difference of ERS score between ERGcluster A and B. (**L**) The difference of ERS score between geneCluster A and B.

For further quantifying the ERS state for a single sample, all samples from the TCGA, GSE28735, GSE62452, GSE57495 and GSE85916 datasets were combined and then randomly grouped into training and verification cohorts using a ratio of 5:5. Then LASSO and multivariate Cox regression analysis were performed to establish a three-gene ERS-related model utilizing the ERS-related DEGs (Figure 5A–5C). Survival analysis showed that in the training, validation and whole cohort, the survival time of patients with a low ERS score was remarkably longer than those with a high ERS score (Figure 5D–5F). In addition, Kaplan–Meier survival curves indicated that high expression of MET, MUC16, and KRT7 were related to worse survival in PC (Figure 5G–5I). Alluvial diagram indicated the low ERS score group mainly corresponded to ERGcluster A and geneCluster B, while high ERS score patients mostly corresponded to ERGcluster B and geneCluster A (Figure 5J). The ERS score of patients in ERGcluster A was remarkably lower compared to ERGcluster B (Figure 5K), and the ERS score of patients in geneCluster B was remarkably lower compared to geneCluster A (Figure 5L). We then examined the relationships between the model genes and ERGs, and the findings revealed that model genes were remarkably positively correlated with most ERGs, while negatively correlated with SCAMP5, PLA2G6, CLU and BRSK2 (Supplementary Figure 5). The association between the ERS score and the clinicopathological characteristics of PC was assessed in this study. The findings demonstrated that at high pathological grade, ERS score was remarkably higher (Supplementary Figure 6). High TNM stage patients had ERS score that were higher than low TNM stage patients, but the difference was not statistically significant, which may have been caused by the small sample size (Supplementary Figure 7). Additionally, ERS score was found to be an independent risk factor for a poor outcome in PC by univariate and multivariate Cox regression (Supplementary Figure 8).

### GSVA, GSEA and immune analysis

**Figure 6:**
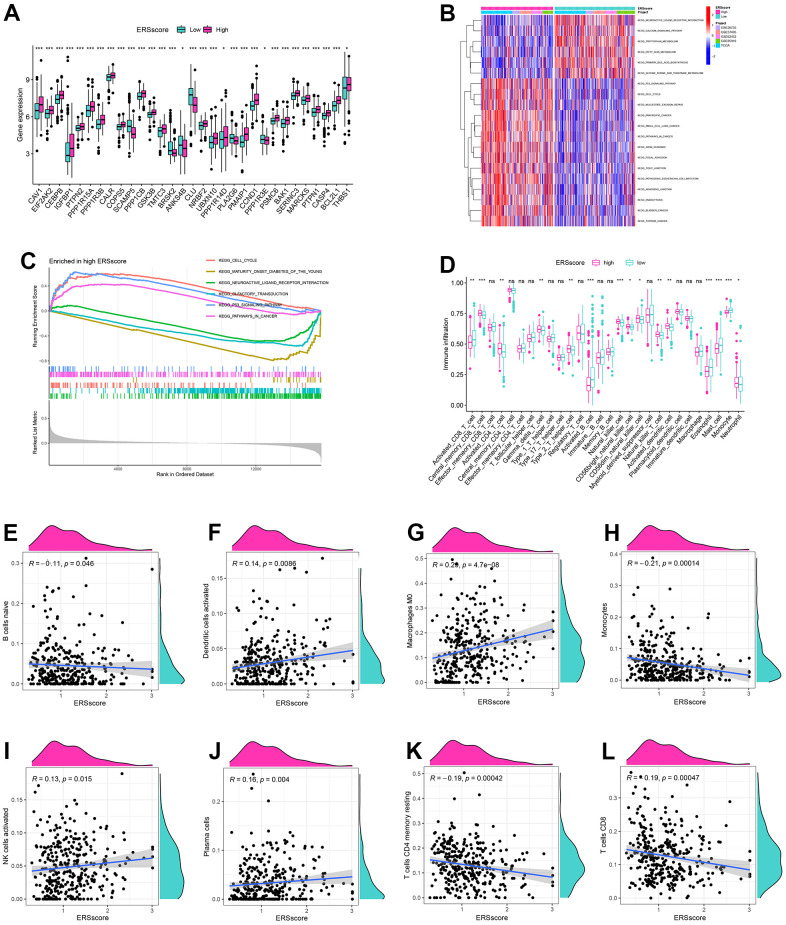
**GSVA, GSEA and immune analysis.** (**A**) The expression difference of endoplasmic reticulum stress-related genes between high and low ERS score. (**B**) GSVA analysis. (**C**) GSEA analysis. (**D**) ssGSEA analysis. Correlation of naive B cells (**E**), activated dendritic cells (**F**), macrophages M0 (**G**), monocytes (**H**), activated NK cells (**I**), plasma cells (**J**), memory resting CD4+ T cells (**K**) and CD8+ T cells (**L**), and ERS score based around the CIBERSORT algorithm. (*p<0.05; **p<0.01; ***p<0.001).

Compared to the patients in the low ERS score group, most ERGs were considerably higher expressed in the high ERS score group, which indicated a higher ERS degree in the patients with a high ERS score (Figure 6A). GSVA found that the remarkable enrichment pathways within the low ERS score group included “neuroactive ligand-receptor interaction”, “tryptophan metabolism”, “calcium signaling pathway”, etc. By contrast, “p53 signaling pathway”, “nucleotide excision repair”, “pancreatic cancer”, “cell cycle” and “pathways in cancer” were considerably enriched in the high ERS score group (Figure 6B). GSEA also indicated “p53 signaling pathway” and “cell cycle” were remarkably enriched in the high ERS score group, and “neuroactive ligand-receptor interaction” and “maturity-onset diabetes of the young” were considerably enriched in the low ERS score group (Figure 6C).

To identify the relationship of the tumour microenvironment and ERS-related model, ssGSEA and CIBERSORT algorithms were carried out. The ssGSEA found remarkably higher infiltration score in activated B cells, activated CD8+ T cells, mast cells, and monocytes within the low ERS score group. The levels of infiltration of activated CD4+ T cells, activated DC cells, NK cells, neutrophils, and type 2 T helper cells within the high ERS score group were significantly increased (Figure 6D). CIBERSORT algorithm indicated ERS score was remarkably negatively associated with B cells, monocyte, memory resting CD4+ T cells, and CD8+ T cells, but positively associated with DCs, macrophages M0, and NK cells (Figure 6E–6L).

### Mutation and drug sensitivity analyses

**Figure 7:**
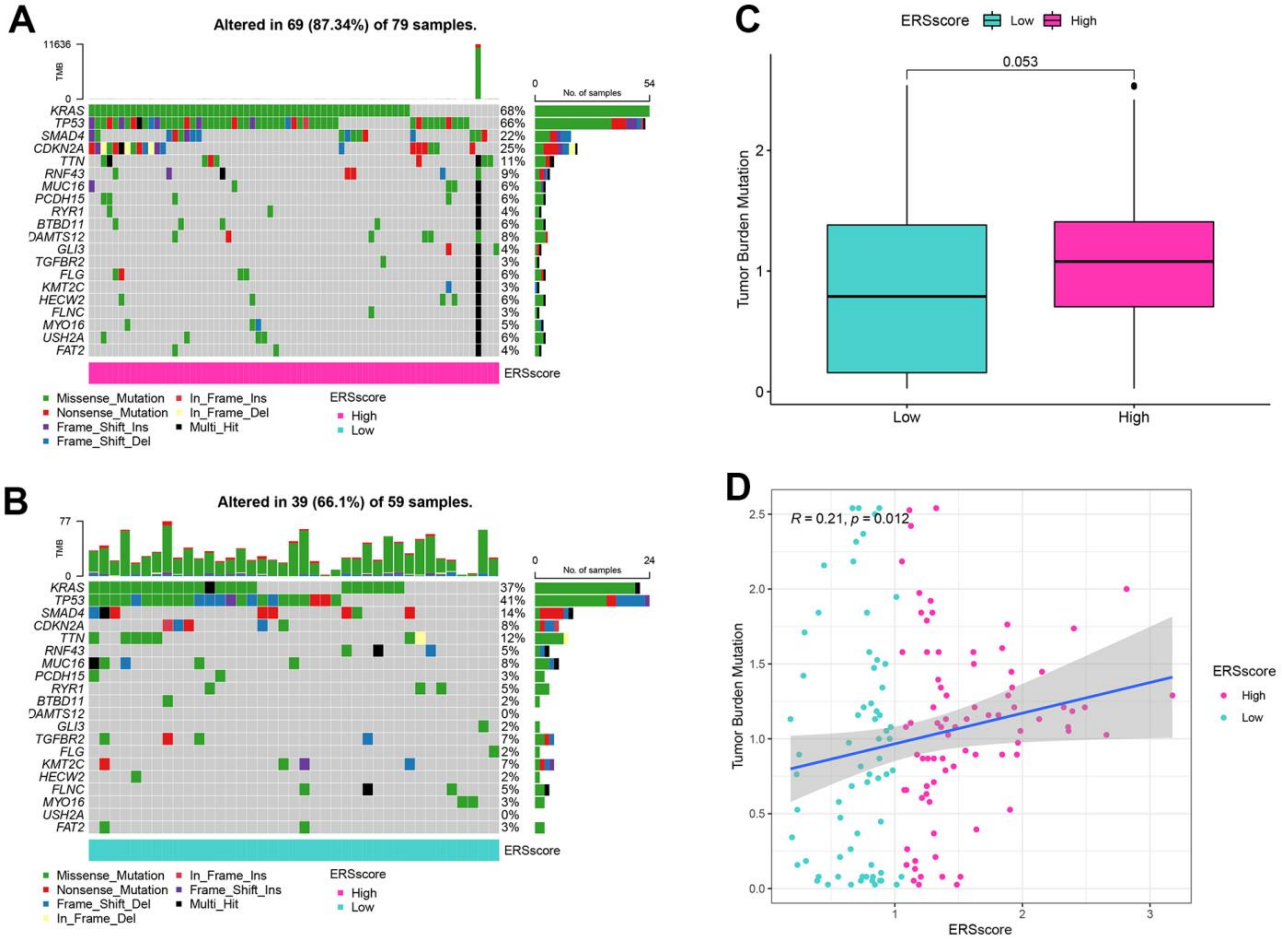
**Mutation analysis.** (**A**) Waterfall map of mutation landscape within the high ERS score group. (**B**) Waterfall map of mutation landscape within the low ERS score group. (**C**) The tumour mutation burden (TMB) between the different ERS score groups. (**D**) Correlation of TMB and ERS score.

Previous studies indicated that inactivated mutations in the tumour suppressor genes TP53, SMAD4, and CDKN2A and activated mutations in the proto-oncogene KRAS were closely related to the incidence as well as the bad prognosis in PC [35, 36]. The genetic mutation landscapes between the different ERS score groups were analyzed in this study. Results found KRAS, TP53, SMAD4, and CDKN2A were the top four mutated genes, and the mutation frequency within the high ERS score group was higher (Figure 7A, 7B). In addition, TMB for every sample was computed, and results showed TMB within the high ERS score group was remarkably higher, as well as the ERS score being considerably positively related to TMB (Figure 7C, 7D).

**Figure 8:**
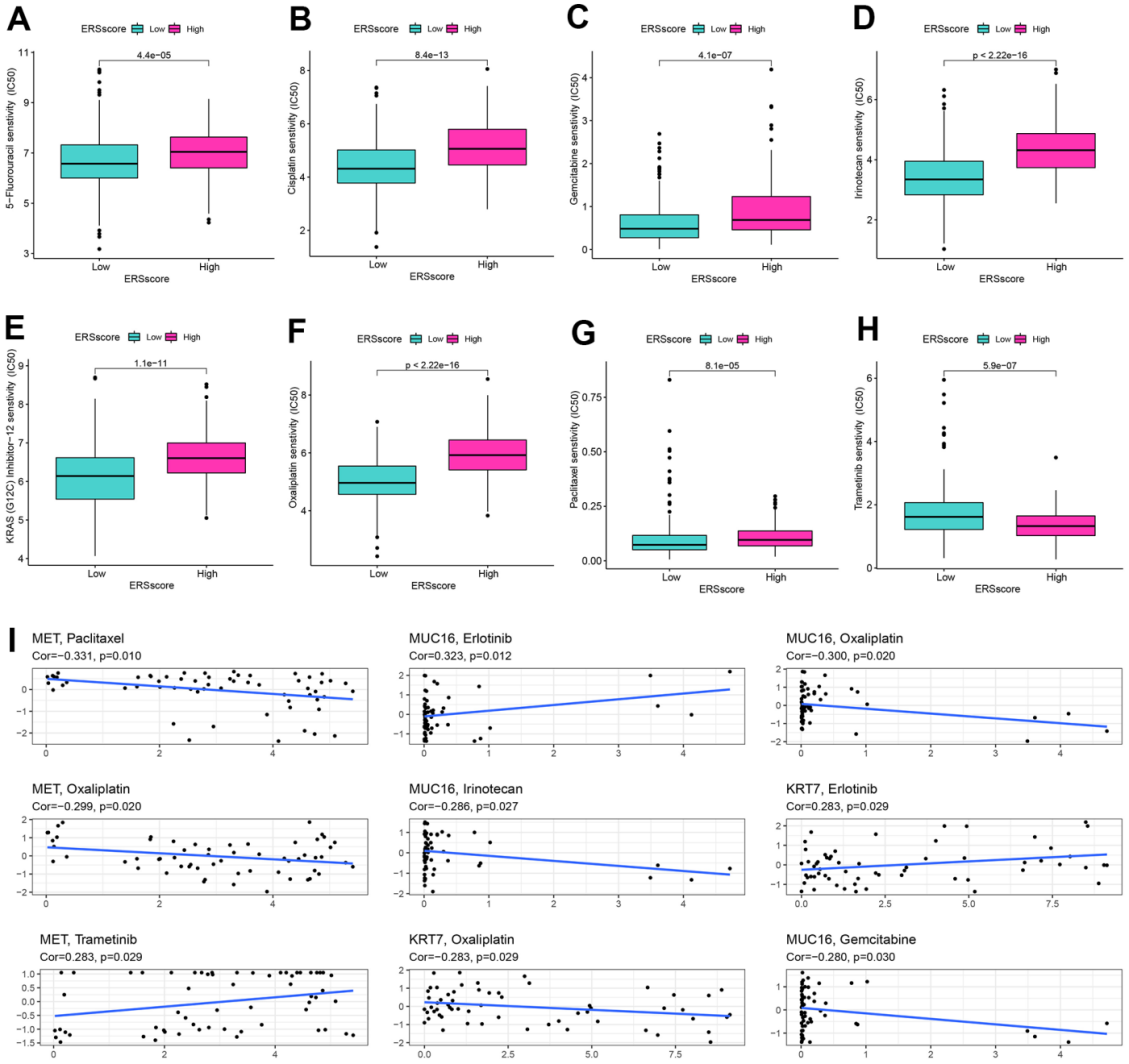
**Drug sensitivity analysis.** The sensitivity of 5-Fluorouracil (**A**), cisplatin (**B**), gemcitabine (**C**), irinotecan (**D**), KRAS (G12C) inhibitor-12 (**E**), oxaliplatin (**F**), paclitaxel (**G**), and trametinib (**H**) between the high and low ERS score groups. (**I**) Correlation of drug sensitivity and MET, KRT7, and MUC16 expression.

Drug-assisted therapy, especially chemotherapy, is vital to improving the survival time for PC. However, the effectiveness of drug therapy is challenged by drug resistance that develops before and during treatment. Clarifying each patient's sensitivity to different drug therapies is critical for clinicians to form personalized treatment plans. The relationship of drug sensitivity and ERS-related model was evaluated through the “oncoPredict” package. Results showed that the low ERS score group had a better response to 5-fluorouracil, cisplatin, gemcitabine, irinotecan, KRAS (G12C) inhibitor-12, oxaliplatin, and paclitaxel, while higher ERS score had better response to trametinib (Figure 8A–8H). Utilizing the CellMiner database, we revealed that MET was remarkably negatively related to the sensitivity of paclitaxel and oxaliplatin, and remarkably positively related to trametinib. MUC16 was considerably negatively related to the sensitivity of oxaliplatin, irinotecan, and gemcitabine, and considerably positively related to the sensitivity of erlotinib. KRT7 was considerably negatively related to the sensitivity of oxaliplatin and positively related to the sensitivity of erlotinib (Figure 8I).

### Gene expression validation and distribution exploration

**Figure 9:**
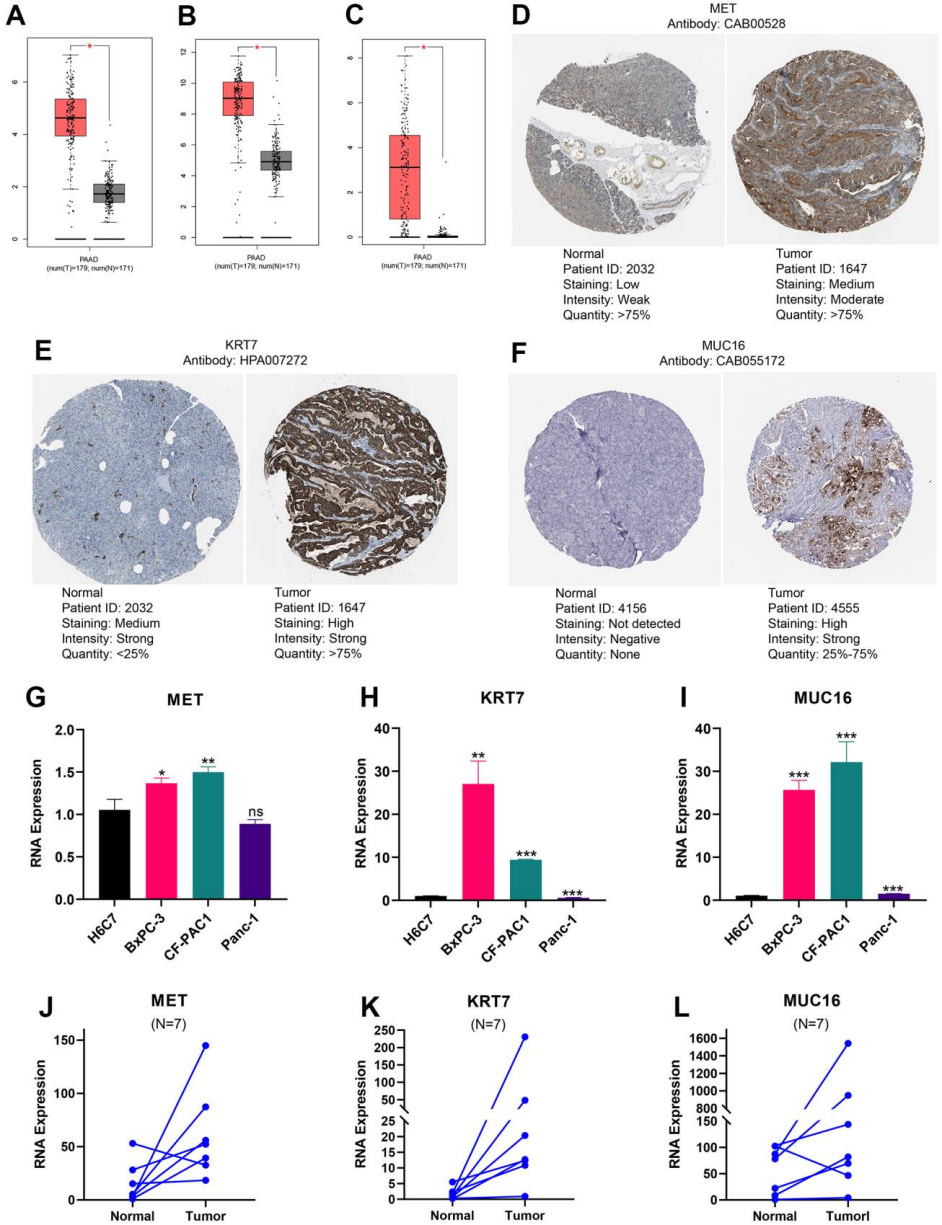
**Expression levels of MET, KRT7, and MUC16.** The expression of MET (**A**), KRT7 (**B**), and MUC16 (**C**) at RNA level. Immunohistochemical images of MET (**D**), KRT7 (**E**), and MUC16 (**F**). The expression differences of MET (**G**), KRT7 (**H**), and MUC16 (**I**) between pancreatic ductal epithelium and pancreatic cancer cell lines. The expression difference of MET (**J**), KRT7 (**K**), and MUC16 (**L**) between pancreatic tumour and adjacent tissue. (*p<0.05; **p<0.01; ***p<0.001).

Based on GEPIA database, we found that mRNA expression of MET, KRT7, and MUC16 was remarkably higher in pancreatic tumour than in normal tissues (Figure 9A–9C). We downloaded immunohistochemical images of pancreatic tumour tissue and normal tissue through HPA platform, and found the protein expression of MET, KRT7, and MUC16 in pancreatic tumour were higher than normal tissues (Figure 9D–9F) (Figure 9D is available from the following URL: https://www.proteinatlas.org/ENSG00000105976-MET/tissue/pancreas#img, https://www.proteinatlas.org/ENSG00000105976-MET/pathology/pancreatic+cancer#img; Figure 9E is available from the following URL: https://www.proteinatlas.org/ENSG00000135480-KRT7/tissue/pancreas#img, https://www.proteinatlas.org/ENSG00000135480-KRT7/pathology/pancreatic+cancer#img; Figure 9F is available from the following URL: https://www.proteinatlas.org/ENSG00000181143-MUC16/tissue/pancreas#img, https://www.proteinatlas.org/ENSG00000181143-MUC16/pathology/pancreatic+cancer#img). What’s more, we validated the expression of MET, KRT7, and MUC16 using RT-qPCR through *in vivo* and *in vitro* experiments. Compared to HPDE6-C7, MET was significantly higher expressed in PC cell lines BxPC-3, CF-PAC1 (Figure 9G), KRT7 was significantly higher expressed in PC cell lines BxPC-3, CF-PAC1 (Figure 9H), and MUC16 was significantly higher expressed in PC cell lines BxPC-3, CF-PAC1, and Panc-1 (Figure 9I). Compared to pancreatic normal tissues, the expression levels of MET, KRT7, and MUC16 were remarkably higher in pancreatic tumour tissues (Figure 9J–9L).

**Figure 10:**
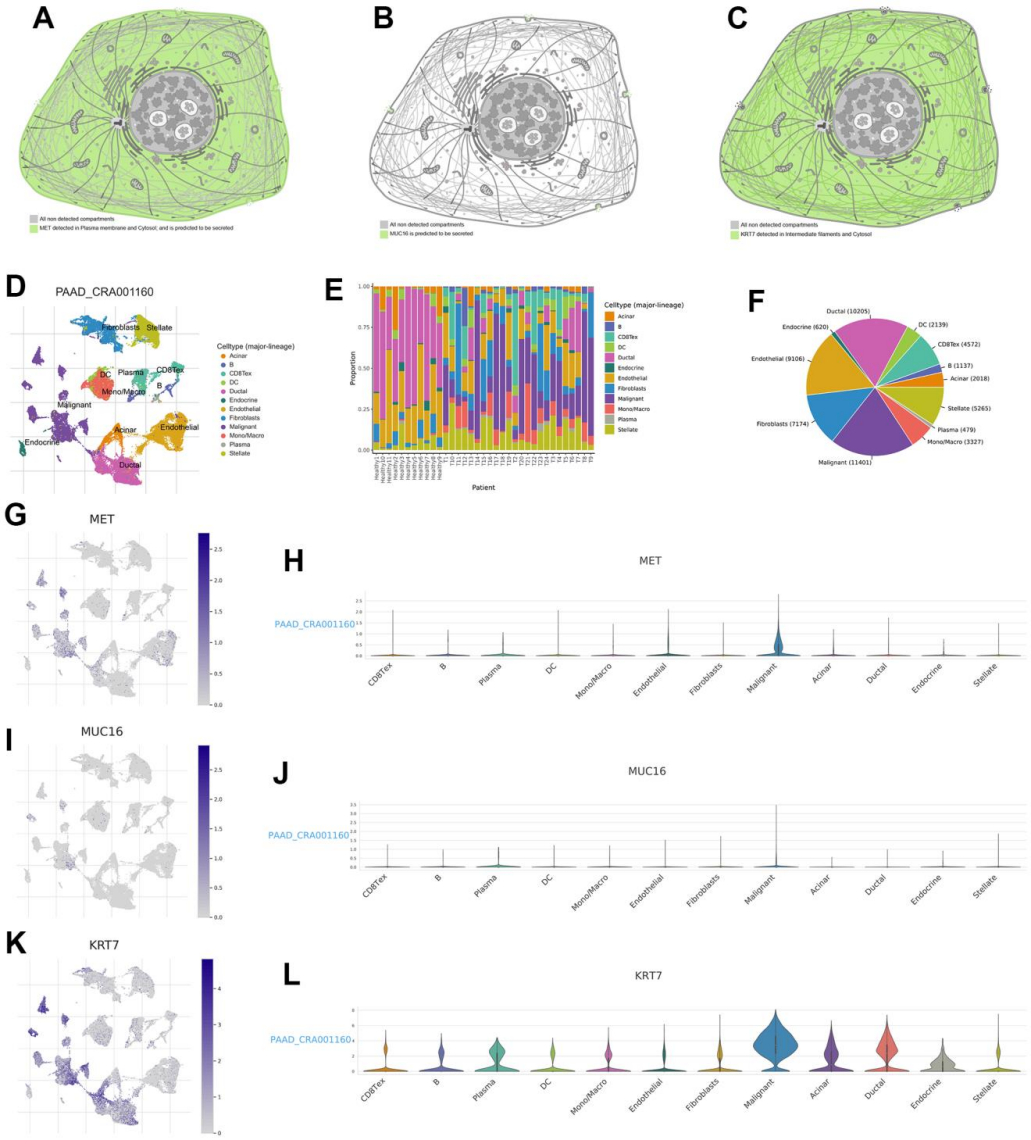
**Expression distribution of MET, MUC16, and KRT7 and single-cell analysis.** The expression distribution of MET (**A**), MUC16 (**B**), and KRT7 (**C**) in different cell substructures. (**D**) Annotation of each cell subset. (**E**) The proportion of each cell subset in each sample. (**F**) The percentages of each cell in pancreatic tumour microenvironment. (**G**, **H**) The distribution and proportion of MET expression within each cell subset. (**I**, **J**) The distribution and proportion of MUC16 expression in each cell subset. (**K**, **L**) The distribution and proportion of KRT7 expression in each cell subset.

We further explored the distribution of gene expression in different cell substructures as well as different cell types. MET was detected in the plasma membrane and cytosol and was predicted to be secreted out of the cell (Figure 10A, the URL is https://www.proteinatlas.org/ENSG00000105976-MET/subcellular). MUC16 was predicted to be secreted out of the cell (Figure 10B, the URL is https://www.proteinatlas.org/ENSG00000181143-MUC16/subcellular). KRT7 was detected in intermediate filaments and the cytosol (Figure 10C, the URL is https://www.proteinatlas.org/ENSG00000135480-KRT7/subcellular). Unlike the gene expression data obtained from conventional tissue RNA-seq, single-cell RNA-seq may offer transcription data from a single cell. The single-cell analysis was performed using the single-cell dataset CAR001160 through TISCH platform. Results revealed that in the tumour microenvironment of PC, malignant, duct, endothelial and stellate cells were the top four cell types with the highest proportion (Figure 10D–10F). Compared to other cell subtypes in the tumour microenvironment, the expression of MET, MUC16, and KRT7 was higher in pancreatic malignant cells (Figure 10G–10L).

## DISCUSSION

PC is an extremely malignant digestive tract tumour with a 5-year survival rate of just 10% [37]. Even though the therapy for malignancies has undergone significant evolution in the last few decades, the prognosis of PC has not been distinctly improved. It is unquestionably a long and difficult task to explore the pathologic process of PC and uncover potential treatment strategies. Tumorigenesis, as a pathophysiological process with extremely complicated mechanisms, involves multi-level reactions and mutation accumulation, which are related to a variety of internal and external factors, such as genetic factors, environmental exposure, dietary habits, and so on. As the largest organelle in eukaryotic cells, endoplasmic reticulum is involved in protein synthesis, folding, and transport [38]. Many adverse factors, such as hypoxia, nutritional deficiency, oxidative stress, oncogene activation, and calcium imbalance, can interfere with the normal protein folding process in endoplasmic reticulum, leading to the aggregation of unfolded or misfolded proteins within the endoplasmic reticulum, thus inducing ERS [39–41]. Relevant studies have shown that ERS is involved in multiple mechanisms of tumorigenesis and progression, including tumour formation and metastasis, angiogenesis, chemotherapy resistance, and immune escape [42–44]. However, majority of ERGs have not been investigated in PC. In this study, we established the ERS-related molecular subtypes as well as a prognostic model for PC.

Based on consensus clustering analysis, all the PC patients were classified into ERGcluster A and B. ERGcluster B with a higher ERS level had a significantly worse prognosis. In malignant tumours, cancer cells are in vigourous activity of proliferation and division, facing more oxygen and nutrient deficiency, DNA damage, and other stress stimuli, which makes cancer cells have a more active ERS level [45]. GSVA analysis showed that compared with ERGcluster A, ERGcluster B was significantly enriched within malignant tumor-related pathways like “cell cycle”, “p53 signaling pathway”, “pancreatic cancer”, etc. Immune escape is one of the most important mechanisms in malignant tumour formation. Tumour cells may escape the recognition of the immune system depending on whether they create “self” markers or have sufficient immunosuppressive ability in their microenvironment so as to survive and grow continuously [46]. Previous studies showed that ERS in tumour cells not only participates in regulating intracellular events such as cell proliferation, but may also be involved in the regulation of extracellular effects such as modulating the tumour immune microenvironment and immune responses [47, 48]. Cubillos-Ruiz et al. [19] found that XBP1, an ERS response factor, can restrain the anti-tumour immune response and promote neoplasm endogenous growth. Study from Liu et al. [49] showed that ERS could cause hepatocellular carcinoma cells to release exosome miR-23a-3p, which mediated macrophages to overexpress programmed death ligand 1, thereby inhibiting the anti-tumor immune function of T cells. A large amount of evidence has confirmed that Tregs, neutrophils, and MDSCs contributed to the establishment of an immunosuppressive tumour microenvironment, which in turn helps tumour cells escape the killing function of the immune system [50–53]. In the current study, ssGSEA analysis revealed considerable variations in pancreatic tumour microenvironment between the different ERS-related patterns. Treg cells, neutrophils, and MDSCs were significantly more infiltrated in ERGcluster B with a more active ERS state. This indirectly indicated that ERS might be related to anti-tumour immunity and involve in the process of tumour cells escaping the killing of immune cells.

The ERS-related prognostic model was further established to quantify the ERS score in each individual patient. ssGSEA indicated Treg cells had significantly higher infiltration scores within the high ERS score group, and higher infiltration of CD8+ T cells and B cells within the low ERS score group. CD8+ T cells can kill tumour cells by secreting large amounts of interferon-γ, tumour necrosis factor α and protease granzyme B. Therefore, CD8+ T cells compete for a very important role in anti-tumour immunity and are considered to be associated with a better prognosis for malignant tumour [54–56]. A large sample study of 12,439 breast cancer patients [57] found that the infiltration of CD8+ T cells in tumour tissue may significantly improve the survival time for breast cancer. The study by Miller et al. [58] found that a higher CD8+ T cell infiltration within the tumour was related to a better prognosis in colon cancer. Wang et al. [59] showed that higher CD8+ T cell infiltration within gastric cancer tissues was related to a improved prognosis. Our study showed CD8+ T cells infiltration levels were significantly higher within the low ERS score group. *In vivo*, B cells are mainly involved in humoral immune response, immunoglobulin secretion, antigen presentation, and T cell immune regulation [60, 61]. However, unlike CD8+ T cells, the function of B lymphocytes in tumours is controversial. On the one hand, some studies found that the infiltration of CD20+ B lymphocytes in tumour tissues played a negative regulatory role in tumour growth, and was relate to improved prognosis and reduced recurrence rates in ovarian cancer and cervical squamous cell cancer [62–64]. On the other hand, B cells with STAT3 activity were revealed to be associated with tumour angiogenesis, thus determining that B cells with STAT3 activity can mediate tumour progression and may be utilized as potential treatment targets for malignant tumour [65]. Dong et al. [66] found that CD19+ B cells within metastatic ovarian cancer tissues were related to worse survival. Lundgren et al. [67] indicated that incremental infiltrations of CD20+ and CD138+ B cells were related to a worse prognosis for epithelial ovarian carcinoma. The different roles of B cells in anti-tumor response may depend on different B cell subtypes with distinct phenotypes and functions, which still need to be explored in future studies.

The ERS-related prognostic model included three genes: MET, MUC16, and KRT7, which were associated with a poor prognosis for PC. MET, a proto-oncogene, is transcribed and translated to form c-Met protein, which is a transmembrane tyrosine kinase that takes part in the occurrence and development of a variety of malignant tumours [68, 69]. Study from Dai et al. [15] found that calcium disturbances in endoplasmic reticulum could induce the conversion of the precursor Met (Pro-Met) into a more stable functional form of c-Met (P190Met^NC^), thereby inhibiting ERS-induced apoptosis in hepatocellular carcinoma by maintaining the high activity of PI3K/Akt and MEK/ERK pathways. Li et al. [70] showed that human induced pluripotent stem cell-derived mesenchymal stem cells (iPS-MSCs) can alleviate ERS, inflammation, and apoptosis in the kidneys of high-fat diet-induced obese mice by activating the hepatocyte growth factor (HGF) /c-Met paracrine signaling pathway. MUC16, also known as CA125, is a type I transmembrane glycosylated protein, which is involved in the growth, proliferation, apoptosis inhibition, chemotherapy resistance, metabolic reprogramming, and immune evasion of tumour cells [71, 72]. MUC16 was detected to be highly expressed in various tumours, like PC [73], ovarian carcinoma [74] and cervical cancer [75], etc. Liang et al. [76] showed that high expression of CA125 in serum was related to a worse prognosis of pancreatic cancer. Our study showed that MUC16 was significantly higher expressed in pancreatic tumour than normal pancreatic tissues and was related to a worse prognosis. Keratin 7 (KRT7), as a member of the keratin gene family, is abnormally expressed in different types of malignant tumours, such as esophageal squamous cell carcinoma [77], colorectal cancer [78] and ovarian cancer [79]. The current study found KRT7 was remarkably overexpressed in pancreatic tumours and related to a worse prognosis, suggesting KRT7 might function as a possible therapeutic target for PC.

Chemotherapy is the main treatment for postoperative and advanced PC patients. At present, the first-line chemotherapy regimen for PC is FOLFIRINOX (fluorouracil, oxaliplatin, irinotecan, and leucovorin) or gemcitabine combined with nab-paclitaxel [5]. However, the response rate of pancreatic cancer patients to chemotherapy is not high, and chemoresistance is often the cause of chemotherapy failure. ERS is associated with sensitivity to drug therapy for cancer. Wang et al. [80] found that apatinib could induce ERS-mediated apoptosis and autophagy through the IRE-1α/AKT/mTOR pathway in esophageal squamous cancer, and make tumour cells more sensitive to paclitaxel. Huang et al. [81] showed that reticulocalbin-1 gene knockout can promote ERS-induced apoptosis and make nasopharyngeal carcinoma cells more sensitive to doxorubicin. We study revealed that patients with a low ERS score had a better response to 5-fluorouracil, gemcitabine, irinotecan, oxaliplatin, and paclitaxel, while the high ERS score group had a better response to the targeted drug trametinib. These are instructive for the individualization of patients with PC.

In the current study, we first clarified that ERGs compete for important roles in the carcinogenesis and progression of PC through comprehensive bioinformatics analysis and *in vivo* and *in vitro* experiments. However, there are also some limitations that could be improved in future studies. Firstly, survival information for PC was acquired from online databases. Although we used different databases to validate our prognostic model, prospective large clinical samples are still necessary to further confirm the reliability of the model. Secondly, we utilized *in vivo* and *in vitro* experiments to validate the expression of the genes in our panel, but the specific mechanisms of ERS in inducing PC development and interfering with the tumour immune microenvironment still need to be investigated in more in-depth experiments in the future.

## CONCLUSIONS

We first evaluated the genetic variations of ERGs in pan-cancer, and constructed the ERS-related molecular subtypes and prognostic model for PC by extensive bioinformatics analysis and *in vitro* and *in vivo* experiments, which revealed ERS was closely associated with the prognosis, tumour immune microenvironment, genomic mutation, and drug sensitivity of PC. These results will contribute to the further study of molecular mechanisms and novel therapeutic strategies for PC in the future.

## Supplementary Material

Supplementary Figures

Supplementary Table 1

Supplementary Table 2
