# Supplementary material for: Identifying endoplasmic reticulum stress-related molecular subtypes and prognostic model for predicting the immune landscape and therapy response in pancreatic cancer
Source: Aging (Albany NY). 2023 Oct 9;15(19):10549–79. doi: 10.18632/aging.205094 (PMC10599750; doi:10.18632/aging.205094)
Supplement: Supplementary Figures [file aging-15-205094-s001.pdf]

SUPPLEMENTARY FIGURES

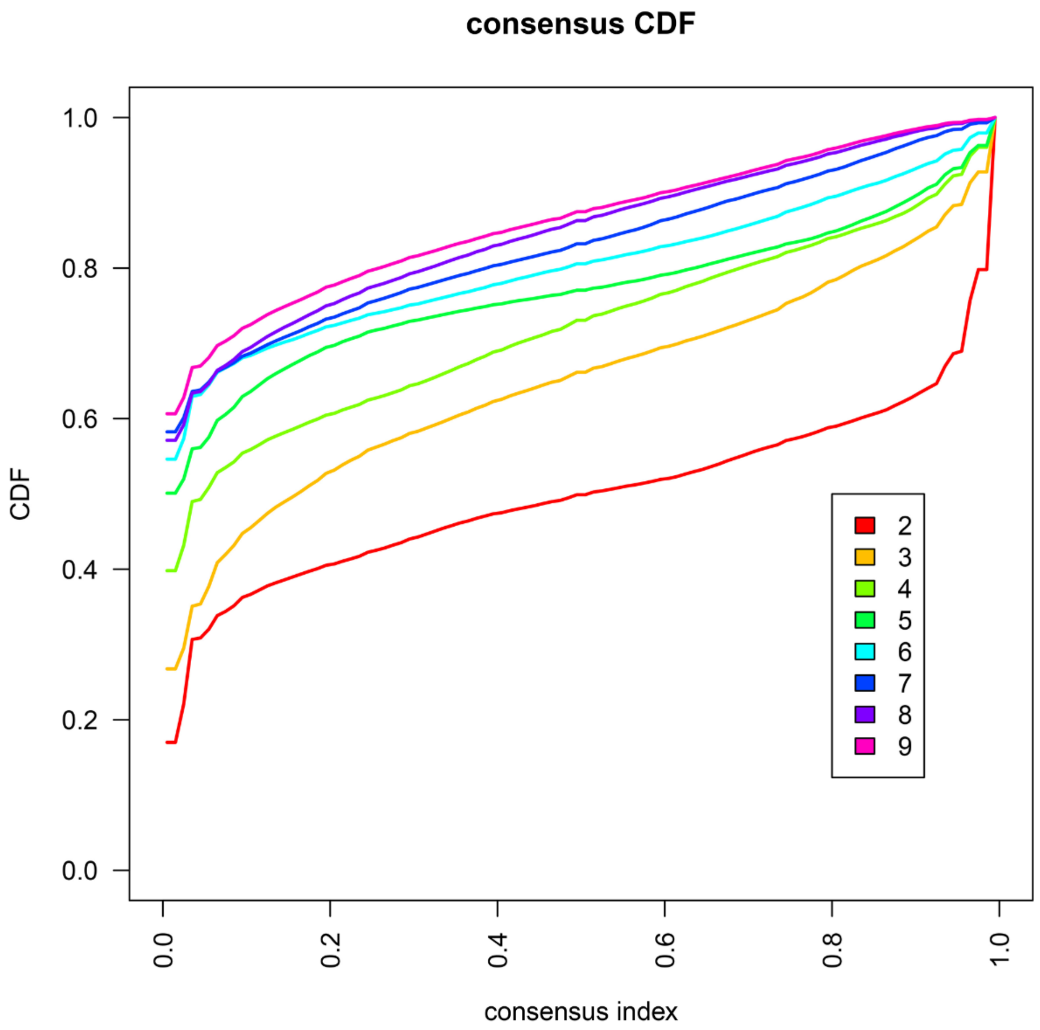

Supplementary Figure 1. Cumulative distribution function curve when cluster number k = 2–9.

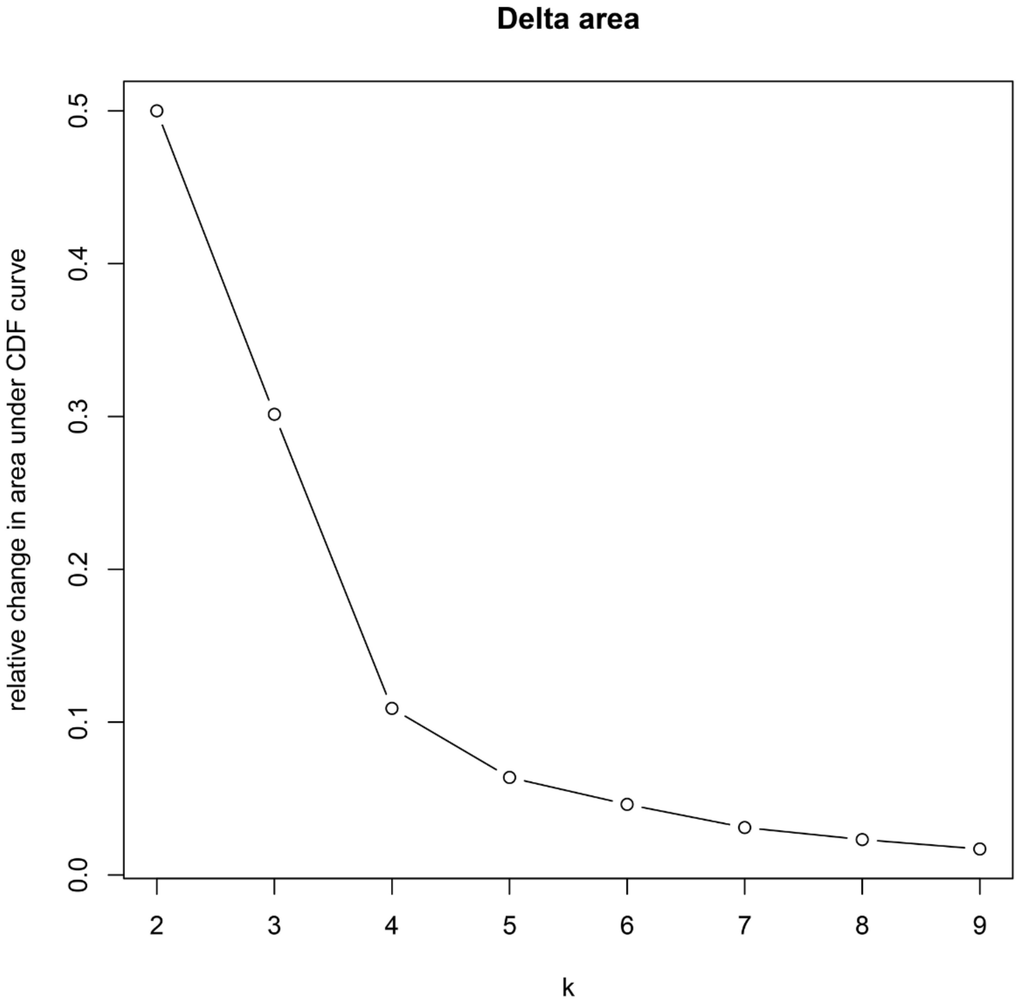

Supplementary Figure 2. Relative change of the area under the cumulative distribution function curve.

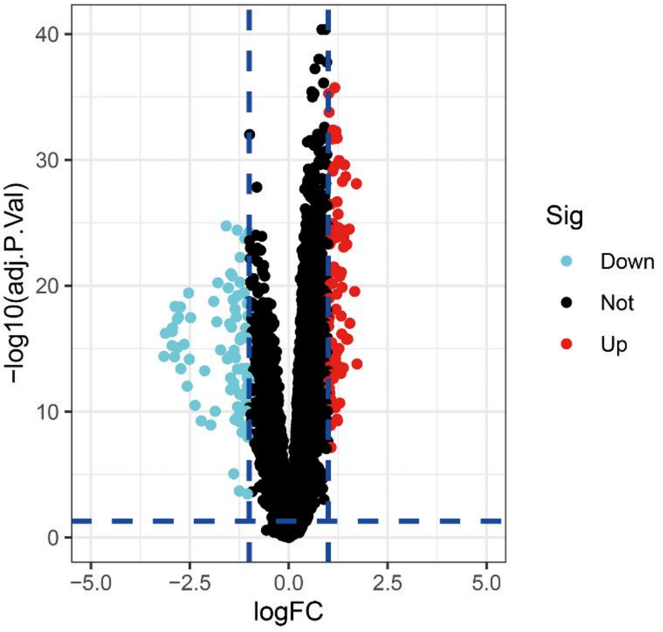

Supplementary Figure 3. The differentially expressed genes between ERGcluster A and B.

	pvalue	Hazard ratio
ANXA1	<0.001	1.357(1.199−1.536)
PLOD2	<0.001	1.493(1.290−1.728)
ARNTL2	<0.001	1.474(1.292−1.682)
TREM1	<0.001	1.269(1.131−1.424)
NT5E	<0.001	1.392(1.240−1.564)
ANLN	<0.001	1.380(1.235−1.541)
PLAUR	<0.001	1.326(1.159−1.516)
CD109	<0.001	1.440(1.265−1.638)
PLAU	<0.001	1.372(1.225−1.535)
FN1	<0.001	1.260(1.138−1.396)
MET	<0.001	1.584(1.394−1.801)
LOXL2	<0.001	1.359(1.207−1.529)
EREG	<0.001	1.310(1.201−1.429)
SLC2A1	<0.001	1.304(1.181−1.440)
DKK1	<0.001	1.187(1.109−1.271)
ADM	<0.001	1.298(1.176−1.433)
FOSL1	<0.001	1.194(1.078−1.322)
CDA	<0.001	1.357(1.229−1.499)
TGFBI	<0.001	1.425(1.261−1.610)
TNFAIP6	0.036	1.118(1.007−1.240)
BEX1	0.002	0.888(0.825−0.956)
AHNAK2	<0.001	1.321(1.194−1.462)
SCEL	<0.001	1.273(1.183−1.370)
EPHA2	<0.001	1.296(1.162−1.445)
PTGS2	0.029	1.093(1.009−1.183)
TMED6	0.025	0.894(0.811−0.986)
ITGA2	<0.001	1.297(1.155−1.456)
CLU	<0.001	0.815(0.728−0.912)
GJB2	<0.001	1.274(1.159−1.399)
CALB2	<0.001	1.202(1.107−1.304)
CDH3	<0.001	1.281(1.164−1.410)
KIF1A	0.009	0.867(0.779−0.965)
LOX	<0.001	1.234(1.097−1.388)
CTHRC1	<0.001	1.209(1.084−1.348)
DCBLD2	<0.001	1.559(1.394−1.743)
GPR87	<0.001	1.183(1.101−1.271)
LAMB3	<0.001	1.282(1.170−1.404)
ADAM12	0.004	1.152(1.047−1.268)
COL12A1	0.010	1.132(1.030−1.244)
INHBA	0.001	1.184(1.067−1.315)
LAMC2	<0.001	1.314(1.198−1.441)
GPRC5A	<0.001	1.235(1.126−1.353)
SULF1	<0.001	1.214(1.093−1.349)
LAMA3	<0.001	1.274(1.159−1.401)
SERPINB5	<0.001	1.194(1.110−1.285)
COL11A1	0.002	1.111(1.040−1.186)
POSTN	0.002	1.154(1.054−1.264)
IGF2BP3	<0.001	1.302(1.176−1.442)
ABCC8	0.008	0.905(0.841−0.975)
GREM1	<0.001	1.179(1.078−1.290)
TNS4	<0.001	1.140(1.062−1.225)
THBS2	0.003	1.170(1.053−1.300)
IL1RN	<0.001	1.201(1.090−1.324)
COL17A1	<0.001	1.203(1.126−1.286)
SCGN	0.024	0.929(0.871−0.990)
SERPINE1	0.003	1.161(1.051−1.281)
TRIM29	<0.001	1.176(1.094−1.265)
PADI1	<0.001	1.162(1.091−1.237)
KRT17	<0.001	1.189(1.115−1.269)
KRT19	<0.001	1.340(1.201−1.495)
GALNT5	0.003	1.140(1.046−1.243)
IGFL2	0.003	1.144(1.047−1.250)
FAM83A	<0.001	1.281(1.194−1.374)
FNDC1	0.023	1.105(1.014−1.204)
SFN	<0.001	1.167(1.076−1.266)
MUC16	<0.001	1.227(1.142−1.320)
MMP11	<0.001	1.152(1.067−1.245)
KRT7	<0.001	1.290(1.186−1.402)
KRT6A	<0.001	1.147(1.095−1.202)
COL10A1	0.007	1.112(1.030−1.200)
CST6	<0.001	1.165(1.083−1.252)
IGFBP1	0.012	1.111(1.024−1.206)
TMPRSS4	<0.001	1.168(1.080−1.263)
KLK6	<0.001	1.170(1.097−1.247)
APOH	0.018	0.909(0.841−0.984)
UCA1	<0.001	1.219(1.138−1.304)
SCG3	0.007	0.905(0.841−0.973)
TNNT1	<0.001	1.172(1.080−1.272)
FGFBP1	<0.001	1.165(1.079−1.258)
KRT23	0.002	1.120(1.042−1.204)
NKX2−2	0.002	0.878(0.809−0.952)
MMP12	0.001	1.142(1.054−1.238)
CHGA	0.004	0.916(0.863−0.973)
PCSK1N	<0.001	0.857(0.791−0.929)
NMU	0.002	1.132(1.046−1.225)
GC	0.036	0.939(0.886−0.996)
PCSK1	0.007	0.892(0.821−0.969)
CHGB	0.031	0.941(0.890−0.994)
CCL20	<0.001	1.130(1.054−1.213)
PCSK2	0.012	0.914(0.852−0.980)
CCL18	0.003	1.116(1.038−1.201)
S100P	0.002	1.098(1.033−1.166)
S100A2	<0.001	1.184(1.113−1.261)
CP	<0.001	1.143(1.064−1.229)
CXCL5	<0.001	1.122(1.059−1.188)
KLK7	<0.001	1.119(1.055−1.187)
MSLN	<0.001	1.118(1.049−1.192)
C7	0.014	0.919(0.859−0.983)
SPRR1B	<0.001	1.171(1.106−1.239)

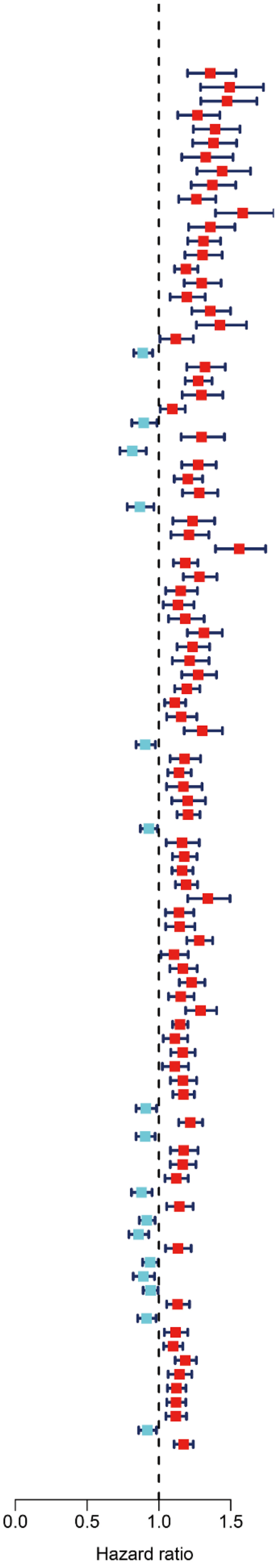

Supplementary Figure 4. Univariate Cox regression analysis using the differentially expressed genes between ERGcluster A and B.

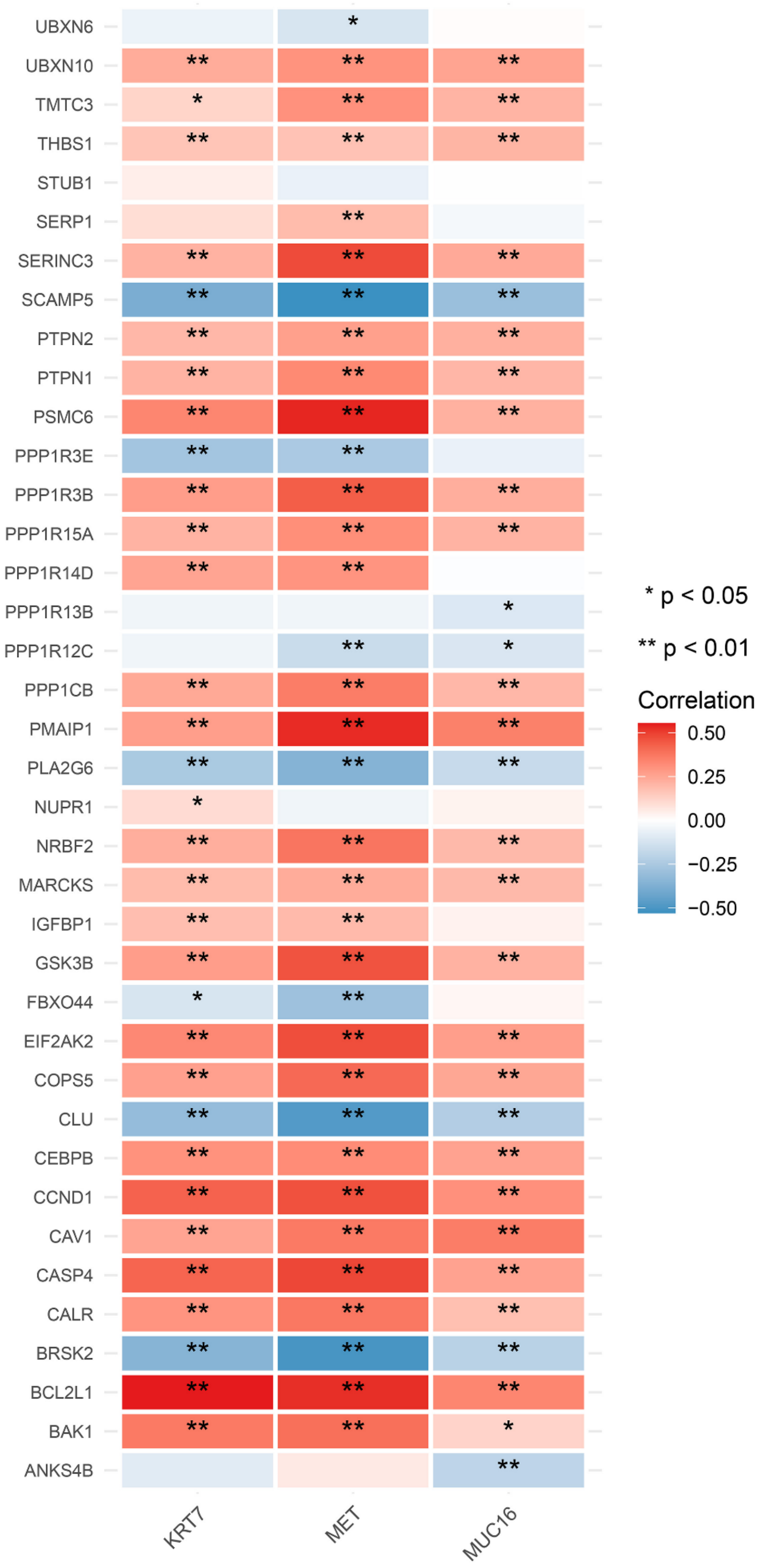

Supplementary Figure 5. The correlation of model genes and ERGs.

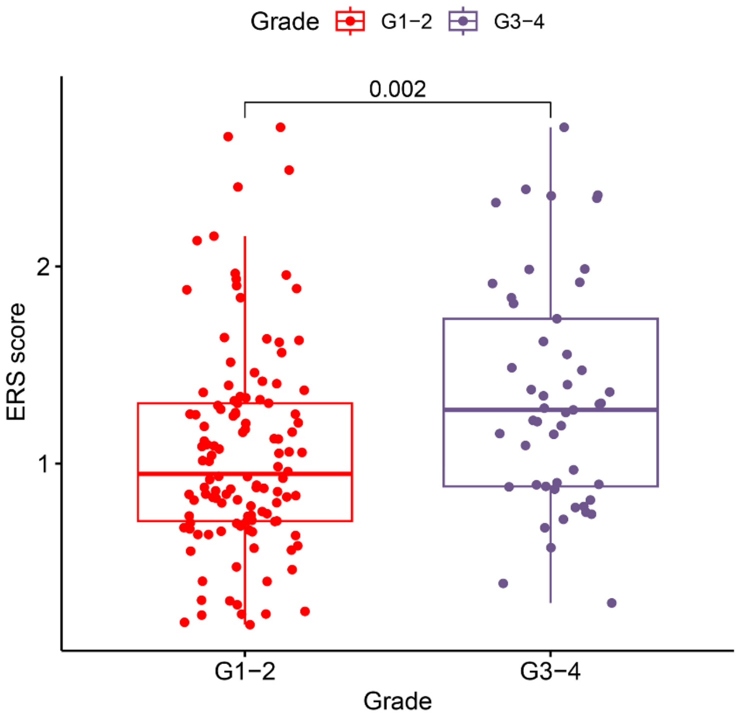

Supplementary Figure 6. The comparison of ERS score between the high and low pathological grade.

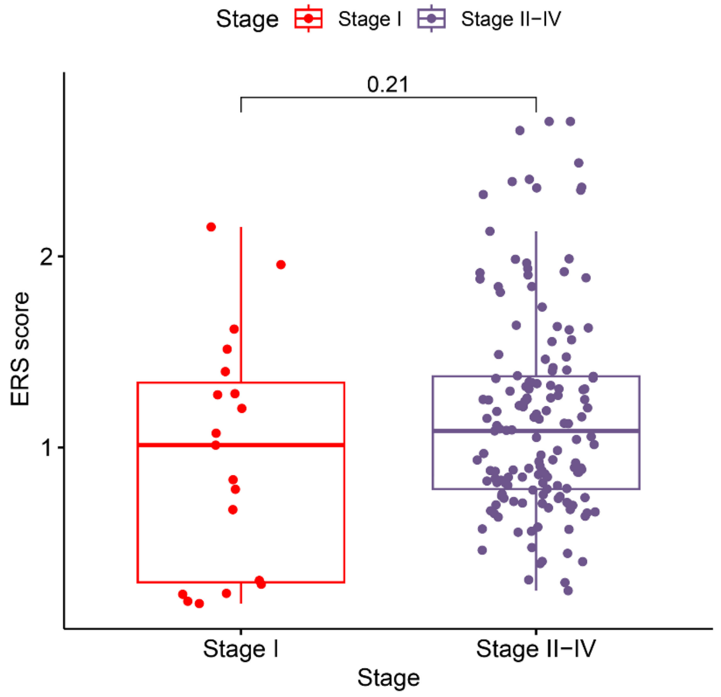

Supplementary Figure 7. The comparison of ERS score between the high and low TNM stage.

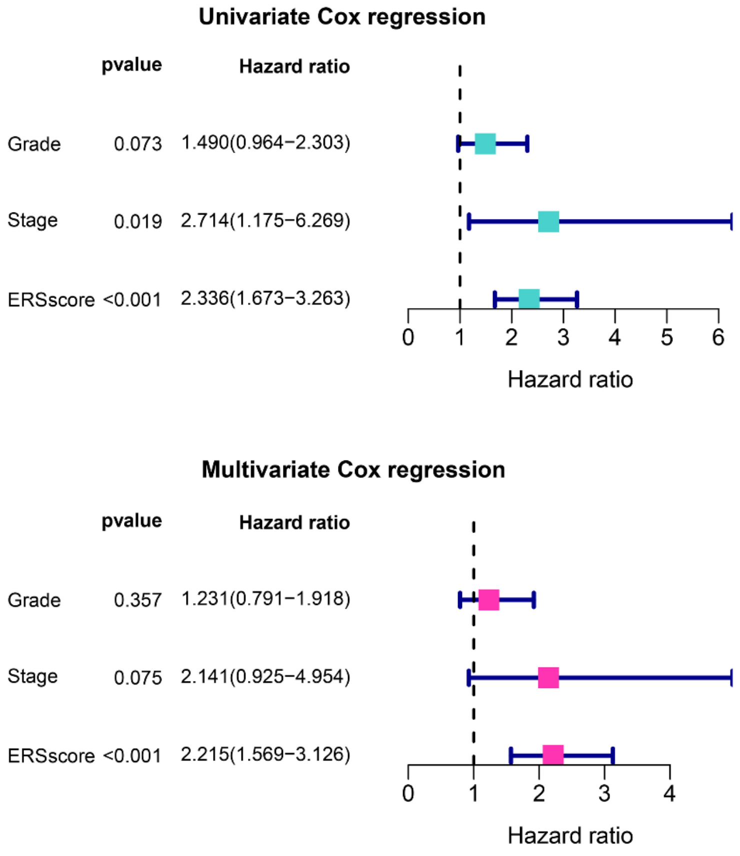

Supplementary Figure 8. Forest maps of univariate and multivariate Cox regression.
